# Is the “Brainwork Intervention” effective in reducing sick leave for non-permanent workers with psychological problems? Results of a controlled clinical trial

**DOI:** 10.1186/s12889-021-10704-0

**Published:** 2021-04-09

**Authors:** Selwin S. Audhoe, Jan L. Hoving, Bonne J. H. Zijlstra, Monique H. W. Frings-Dresen, Karen Nieuwenhuijsen

**Affiliations:** 1 Amsterdam UMC, University of Amsterdam, Coronel Institute of Occupational Health/Research Center for Insurance Medicine, Amsterdam Public Health research institute, PO Box 22700, 1100 DE Amsterdam, The Netherlands; 2 Faculty of Behavioral and Social Sciences, University of Amsterdam, Research Institute of Child Development and Education, Amsterdam, The Netherlands

**Keywords:** Unemployment, Participation, Return-to-work, Sick leave, Vocational rehabilitation, Intervention, Counselling, Psychological problems

## Abstract

**Background:**

Both the presence of psychological problems and the absence of an employment contract are related to long-term sickness absence, prolonged work disability and unemployment. Studies researching the effectiveness of return-to-work interventions on these non-permanent workers, including unemployed and temporary agency workers and workers with an expired fixed-term contract, are lagging behind. Therefore, a return-to-work intervention called “Brainwork” was developed. The aim of this study was to assess the effectiveness of the ‘Brainwork Intervention’ in reducing the duration of sick leave compared to usual care over a 12-month follow-up.

**Methods:**

In a multicenter controlled clinical trial, using a quasi-randomization procedure, we compared the Brainwork Intervention (*n* = 164) to usual care (*n* = 156). The primary outcome was the duration of sick leave. Secondary outcomes were the duration of sick leave starting from Social Security Agency transfer; the proportion of workers returned to work; the number of hours of paid employment during the follow-up period; the degree of worker participation; the level of psychological complaints; and the self-efficacy for return to work. Protocol adherence (Brainwork Intervention) was considered sufficient when at least three of the five protocol steps were followed. Cox regressions, linear and ordinal regression, and Mixed Model analyses were performed.

**Results:**

All 320 participants were analyzed. The Brainwork Intervention resulted in a non-significant reduction of the duration of sick leave compared to usual care (269 days versus 296 days; HR = 1.29; 95% CI 0.94–1.76; *p* = 0.11). For those working (46%) during the 12-month follow-up, the mean number of hours of paid employment was non-significantly higher in the usual care group (682 h versus 493 h; *p* = 0.053). No significant differences were found for other secondary outcomes. Protocol adherence was 10%.

**Conclusions:**

The Brainwork Intervention as performed with a low protocol adherence did not result in a significant reduction of the duration of sick leave compared to usual care. It remains unclear what the results would have been if the Brainwork Intervention had been executed according to protocol.

**Trial registration:**

The Netherlands Trial Register (NTR); NTR3976 (old registration number NTR4190). Registered September 27th 2013.

**Supplementary Information:**

The online version contains supplementary material available at 10.1186/s12889-021-10704-0.

## Background

Psychological problems are highly prevalent in the general and working populations [1–4]. Sickness absence due to psychological problems such as depression, anxiety and stress-related disorders is increasing in many high-income countries, contributing substantially to disability benefits and permanent exclusion from the labor market [5–8]. Only 50% of the workers sick-listed for 6 months or more due to psychological problems return to work (RTW) [8].

Among the labor force, unemployed and temporary agency workers and workers with an expired fixed-term contract are at even greater risk for sickness absence and prolonged work disability due to psychological problems [9–12]. These workers, who do not, or no longer, have an employment contract, also known as non-permanent workers, represent a particularly vulnerable group within the working population [13]. In the Netherlands, non-permanent workers have a three times greater risk of becoming long-term work disabled (> 18 months) compared to workers with a permanent employment contract (employed workers) [14]. Compared to sick-listed employed workers, sick-listed non-permanent workers perceive their health status more negatively and encounter more psychosocial barriers (such as personal problems, debts, addiction, legal proceedings, care issues) for their RTW [10, 11, 15, 16]. Moreover, these workers experience a greater distance to the labor market compared to sick-listed employed workers as there is no workplace to return to when sick-listed [15].

Relatively more workers have become non-permanent workers over the last decade, due, in part, to the worldwide economic crisis and changing labor market conditions [10, 17–19]. To illustrate the increase in non-permanent workers in the Netherlands, in 2013 more than a quarter of the active labor force was working on a temporary basis, compared to almost 18% in 2001 [20]. Considering the growing rate of non-permanent workers, the increasing rate of sick leave due to psychological problems and the higher risk for prolonged work disability in this group, it is surprising that little attention has been paid to the development of effective RTW interventions for non-permanent workers [21]. A recent randomized controlled trial evaluating the effect of a participatory supportive RTW program in sick-listed non-permanent workers with common mental disorders did not result in a significant shorter duration of sick leave [22]. Another recent study evaluating the influence of an interdisciplinary re-employment program on labor force participation among unemployed persons with common mental health problems also revealed unsuccessful RTW [23]. And a study by Reme et al. [24] assessed the effects of individual placement and support (IPS) in workers with both common and severe mental health problems and with various work statuses. While the IPS model was superior to usual care in terms of work participation, this could not be demonstrated for the unemployed subgroup. As a result, there is a growing need to develop RTW interventions for these workers that address the absence of a workplace to which they can return. Enhancing work participation is important since working even a few hours during the RTW process is a strong predictor for successful full RTW [25] and re-employment after unemployment has been shown to lead to increased mental health [26, 27]. Previous research has also been shown that interventions with a workplace component, including vocational counseling, are more likely to succeed in increasing RTW than interventions that do not include such a component [28–30].

In the Netherlands, the Sickness Benefits Act provides a social security safety net for sick-listed workers that do not have an employment contract. The sick-listed non-permanent worker receives a supportive income for a maximum of the first 2 years of sickness absence. There are no legislative mandates for these workers to be returned to their previous/last job. Since there is no employer or workplace to return to, the Dutch Social Security Agency (SSA) is responsible for sickness absence counseling. Sickness absence counseling of these non-permanent workers is conducted by a team of occupational health (OH) professionals from the SSA. An OH professional team consists of insurance physicians (IPs), vocational rehabilitation counselors, labor experts, secretaries and sometimes a nurse practitioner. Counseling by the SSA and sickness benefit terminates once the IP has determined that full recovery of health and/or full work ability for the last performed work has been achieved, i.e., functional work limitations for the last performed work (with or without actual RTW of the worker) are no longer present. The current sickness absence counseling of non-permanent workers (usual care) is not structured according to a fixed protocol. Furthermore, there is no protocol for the referral to RTW programs. The Brainwork Intervention for sick-listed non-permanent workers with psychological problems was developed by a team of professionals of the SSA with different backgrounds, i.e., IPs, labor experts and vocational rehabilitation counselors [13]. Evidence guided the development of the Brainwork intervention [31]. Prior to the development of the intervention there was awareness of the negative effects of unemployment on mental health which has been extensively described in the literature [9–12]. Secondly, the positive effects of activating approaches [32, 33] and re-employment [26, 27, 34–36] were noted, as well as the importance of a rapid start of sickness absence counseling on shortening the duration of sick leave in non-permanent workers [37]. The newly developed Brainwork Intervention is characterized by an activating approach, which means that in the early stage of sick leave (within 2 to 5 weeks), the workers are stimulated to engage in physical exercise and undertake activities aimed at regaining control and functional recovery while job coaches actively support their search for jobs (temporary or otherwise). Besides the activating approach, the Brainwork intervention contains several other elements such as a protocol-based stepped care approach, category classification of the worker and intensive vocational counseling. The content of the intervention differs and is tailored to the severity of the psychological problems and functional impairments, as well as to the specific psychosocial problems encountered by the sick-listed worker. The interventions were provided by OH professionals in collaboration with external partners (e.g., vocational rehabilitation agencies and mental health institutions/professionals) specialized in addressing the target group. Our hypothesis is that this approach will lead to functional recovery and reduce the sick-leave duration of sick-listed workers [13].

The main aim of this study was to assess the effectiveness of the Brainwork Intervention in reducing the duration of sick leave for non-permanent workers with psychological problems compared to usual care. A secondary aim was to assess the effectiveness of the Brainwork Intervention on the: (1) duration of sick leave starting from SSA transfer; (2) proportion of workers returned to work; (3) degree of worker participation; (4) number of hours of paid employment during follow-up; (5) level of psychological complaints; and (6) self-efficacy for RTW.

## Methods

### Study design and setting

The present multicenter study is a two-armed quasi-randomized controlled clinical trial with a follow-up period of 12 months. This study was carried out in collaboration with three regional offices of the SSA across the Netherlands (east, south-west, south regions). Participants were allocated to two groups: an intervention group that received the Brainwork Intervention and a control group that received usual care. The design and procedures of the study have been described in detail in an earlier publication of the study protocol [31]. This study adheres to the CONSORT reporting guidelines [38].

### Study population

Between January 2014 and September 2014, all newly received sick reports of non-permanent workers from the participating SSA offices who met the inclusion criteria were included in the study until the required number of at least 300 participants was reached. The inclusion and exclusion criteria are listed in Table 1. Mild psychiatric disorders include, for example, adjustment disorder, stress-related disorder, mild depressive disorder, mild anxiety disorder and mild post-traumatic stress disorder. Moderate-severe psychiatric disorders include, for example, anxiety disorder, depressive disorders, post-traumatic stress disorder, somatic symptom disorder and Attention-deficit hyperactivity disorder.

**Table 1:** Overview of inclusion and exclusion criteria

**Inclusion criteria**	
• unemployed or temporary agency worker or worker with an expired fixed-term contract	
• age between 18 and 64 years	
• ^a^sick-listed and not expected to RTW within two weeks after either reporting sick or having contact with the vocational rehabilitation counselor of SSA *(An RTW expectation within two weeks of reporting sick corresponds to Brainwork category classification 0; see “Additional file* *1**”)*	
• mild to moderate-severe psychiatric disorder as main reason for sickness benefit claim	
• adequate command of the Dutch language	
**Exclusion criteria**	
• recent pregnancy or up to three months after delivery	
• substance addiction (alcohol, drugs or medicines) as main reason for sickness benefit claim	
• severe psychiatric disorder with an expected recovery of more than one year, e.g., hospitalization or day treatment *(This corresponds to Brainwork category classification 3; see “Additional file* *1**”)*	

^a^The selection of sick-listed “not expected to RTW (recover) within two weeks” is performed by the nurse practitioner or IP, initially on the basis of the completed self-report SSA-specific questionnaire and if necessary with additional information by telephone contact with the worker

In the regular work process of the SSA, each sick-listed worker (i.e., intervention and control group) receives a segmentation code by either the nurse practitioner, who works under the supervision of the IP, or the IP within 2 weeks after the SSA received the sick report. The code indicates the professionals’ estimated duration for sick leave based on a self-report SSA-specific questionnaire. The questionnaire includes, among other things, topics about health complaints, the tasks the worker cannot perform due to the complaints, RTW expectancy of the worker, information about medical treatment, and whether the worker performs volunteer work. Four segmentation codes can be distinguished: code 1 indicates a sick-leave duration of less than 13 weeks; code 2 a sick-leave duration of between 13 and 52 weeks: code 3 a sick-leave duration of between 52 and 104 weeks; and code 4 indicates no expectancy of recovery or work participation. For our study, segmentation codes 1 and 2 were relevant. These codes correspond to the Brainwork category classification 1 (estimated recovery < 3 months) and 2 (estimated recovery 3 to 12 months). See “Additional file 1″ for an overview of the Brainwork category classification of the worker.

The power analysis obtained using the nQuery Advisor program showed that 144 participants were needed per group (288 total) to detect a mean difference in duration of sick leave of 40 days [31].

The Medical Ethics Committee of the Amsterdam UMC, location Academic Medical Center (AMC), University of Amsterdam approved the study design. The study was listed in the Netherlands Trial Register (NTR) under NTR3976 (old registration number: NTR4190); (see: https://www.trialregister.nl/trial/3976).

### Procedure

#### Participants

An information letter was provided to the workers who were included in the study by the staff IP of the relevant regional SSA office. The letter included information about the study and a request to obtain informed consent for completing questionnaires and linking questionnaires data to routinely collected data during the study. Sick-listed workers who gave informed consent filled out the baseline questionnaire and were sent the follow-up questionnaires by the investigators.

#### Occupational health professionals

At each participating SSA office, a team of OH professionals was appointed as an intervention team and one as a control group team. The intervention teams of the three participating SSA offices included 10 (three IPs), 12 (three IPs) and 10 (four IPs) professionals respectively. The number of professionals in the control teams was comparable to that of the intervention teams. All of the OH professionals of the intervention team received instruction and coaching sessions. A two-day training course in motivational interviewing was part of the coaching. The professionals were taught the motivational interviewing skills necessary to activate the sick-listed workers’ participation in the Brainwork Intervention, to initiate positive behavioral changes and to address sick-listed workers’ resistance to change. The intervention teams received instructions not to share any information about the intervention with the control group teams.

### Interventions

#### Brainwork intervention

The rationale of the Brainwork Intervention, the Brainwork category classification of the worker and an overview of the Brainwork Interventions per category have been described in detail elsewhere [13, 31]. These are included as “Additional files 1 and 2”. Briefly, the Brainwork Intervention is designed to assist non-permanent workers who are sick-listed due to psychological problems with their RTW. Within five working days of the SSA receiving the sick report, a face-to-face contact takes place at the SSA between the OH professional and the sick-listed worker. The customized content of the intervention differs depending on the severity of the psychological problems and the specific psychosocial problems the sick-listed worker needs to address (see Additional file 2). The components of the intervention, provided by vocational rehabilitation agencies and mental health institutions/professionals outside the SSA, can include an exercise program, vocational training, gym membership and attention tailored to their mental and/or psychosocial problems (e.g., dealing with coping problems or eye movement desensitization and reprocessing (EMDR) for persons with impaired trauma counseling). All interventions are combined with counseling by vocational rehabilitation agencies where job coaches, labor experts and vocational rehabilitation counselors specialized in searching for and reintegration into suitable work, provide intensive RTW guidance to the sick-listed worker. All with the aim of achieving reintegration into new primary paid work or finding a suitable workplace (with possible view to paid work) to enhance work experience and/or regaining control. Based on the category classification, explicit goals and timetables for recovery were formulated [31].

#### Usual care

The control group received counseling according to care as usual in the SSA setting. Usual care consisted of minimal involvement on the part of the IP (one or two patient contacts in a year) and slightly more intensive contact with other OH professionals. Usually, active sickness absence counseling starts later compared to the Brainwork intervention, ranging from a few weeks to 6 months after reporting sick. An SSA file search in 2008 showed that it took an average of 10 weeks before the first contact of the sick-listed worker with the IP occurred [37]. Furthermore, it was found that a late start (≥8 weeks) of the sickness absence counseling, a late first IP assessment (≥10 weeks) and fewer IP assessments (< 1 contact in 12 weeks) during the sick-leave period were associated with a longer duration of sick leave. Due to the absence of a protocol for referral to RTW programs, the use of RTW interventions remains limited in usual care**.** In contrast to the Brainwork Intervention group, in usual care, early reintegration into primary paid work or enhancing work experience is not an explicit goal as there is no employer or workplace to return to. The main tasks of the IP in usual care are to evaluate the sickness benefit claim of the sick-listed worker and the workers’ fitness for work. The main tasks of other OH professionals are to monitor the sick-listed worker, e.g., to check if the worker is complying with the rules of the Sickness Benefits Act by seeking medical treatment for his complaints and if the symptoms of the worker are improving. The interventions received by the workers in the usual care group were registered.

### Outcome measures

#### Data collection

Data regarding sickness benefit duration, proportion of workers returned to work, paid employment during follow-up and degree of participation are continuously registered by the SSA and were routinely collected from the computerized SSA database. We used data from a follow-up period of 12 months after the date on which the SSA received the sick report. Data regarding psychological complaints and self-efficacy for RTW were collected from self-reported questionnaires at baseline, and 4, 8 and 12 months after the SSA received the sick report. Two reminder letters to complete the questionnaires were sent by mail to to the participants’ home addresses with an interval of 2 weeks. Data entry of the self-reported data was performed by a research assistant using a unique code for each participant.

#### Primary outcome

The primary outcome measure was duration of sick leave and operationalized as duration of the sickness benefit period (in calendar days) from the first day of reporting sick until the termination of the sickness benefit. The sickness benefit ends after a full RTW (e.g., for temporary agency workers) or if the participant is declared fit for work by the IP (e.g., for unemployed workers).

#### Secondary outcomes

The secondary outcome measures were duration of sick leave starting from SSA transfer, the proportion of workers returned to work, number of hours of paid employment during follow-up, degree of participation, psychological complaints, and self-efficacy for RTW. The duration of sick leave starting from SSA transfer is operationalized as the actual duration (in calendar days) that the sick-listed worker was under counseling by the regional office of the SSA until the termination of the sickness benefit. This secondary outcome is of particular importance in workers with an expired fixed-term contract (those whose contract expired while they were sick-listed), because for these workers the difference between total sick leave period (primary outcome) and duration of counseling by the SSA can be more than 1 year. This is due to the fact that sickness absence counseling for workers with an expired fixed-term contract starts at a later time point during the sick leave process than the unemployed and temporary agency workers because the contract workers have an employer at the time of reporting sick. The proportion of workers returned to work was operationalized as the proportion who ended sickness benefit claims and was measured at 12 months after the date that SSA received the sick report. The degree of participation was coded in the ordered categories of: no participation, non-paid work (volunteer work or working in a work experience situation) and paid work, consecutively.

Psychological complaints were measured using the Dutch translation of the General Health Questionnaire-12 (GHQ-12) [39]. The GHQ-12 is one of the most common mental health tools in use and a well-established screening instrument designed to detect non-psychotic psychiatric disorders in people in community and medical settings. It is a 12-item self-report questionnaire concerning the respondent’s assessment of his or her present mental health state. Each item is rated on a four-point response scale. Using the scoring method (0–1–2–3), the sum score ranges from 0 to 36. Low scores reflect better mental health. **‘**Self-efficacy for RTW’ was measured using a validated 11-item RTW self-efficacy questionnaire, with response categories on a 6-point scale [40]. Participants were asked to respond to statements about their jobs, imagining that they would start working their full contract hours again the following day (in their present emotional state/state of mind). In a pilot study of workers on sick leave due to common mental disorders, this questionnaire had a satisfactory construct validity and good reliability [40]. A mean score across the 11 items was used to compute the scale score. The scale score ranges from 1 to 6. Higher scores reflect higher self-efficacy levels.

### Randomization and blinding

Within each of the three participating regional offices of the SSA participants were allocated to the Brainwork Intervention team or usual care team using quasi-randomization, based on sequence of sick-leave reports to the SSA, alternating between intervention and usual care teams in blocks of five workers. The allocation procedure has been described in detail elsewhere [31]. To ensure equal distribution of the different types of workers in the intervention team and usual care team, the sick-listed workers were pre-stratified based on the type of worker (i.e., unemployed and temporary versus expired fixed-term contracts). Equal distribution of the types of workers in both teams was important as the starting point of the intervention was different for the subgroups of workers. The person responsible for allocation of the worker to the teams was unaware of the worker’s characteristics, including the type or severity of the psychological problem.

The participants, OH professionals and intervention partners such as vocational rehabilitation agencies and mental health institutions/professionals were not blinded to the allocation result.

### Protocol adherence

#### Timespan

The following five protocol steps executed by OH professionals were used as process measures for adherence to the protocol: (1) telephone contact by the OH professional with the worker within 2 days of the SSA receiving the sick report; (2) face-to-face contact between the vocational rehabilitation counselor and the worker within five working days of the SSA receiving the sick report; (3) bilateral consultations between the vocational rehabilitation counselor and IP within 2 days after the face-to-face contact with the worker; (4) consultation of the IP, within one to 2 weeks of the bilateral consultations; (5) timely start (due to timely referral) of the intervention within eight working days after consultation of the IP.

Participation in the activities at the above mentioned time points is, due to the Sickness Benefits Act, mandatory to the sick-listed worker, but often provided much later or not even in case of the intervention. Therefore, the adherence to the protocol was considered sufficient when three of the five protocol steps were followed within the given time frame and with the start of the intervention being timely in all cases. Process measures data including the received interventions are registered by the SSA and were collected from the SSA database.

### Statistical analyses

Using the intention-to-treat principle all analyses were conducted at worker’s level. To determine whether the quasi-randomization was performed successfully, descriptive statistics were used to compare the baseline characteristics of intervention and control group. The main analyses were adjusted for prognostic dissimilarities if needed. For those five aspects (process measures) of the protocol which were fixed for all participants, the protocol deviations were analyzed as preparation for the per-protocol analyses. The intention was to compare the results of the intention-to-treat analyses with the per-protocol analyses to assess the presence of bias due to protocol deviations.

Cox regressions analyses were performed to determine hazard ratios (HR) between the intervention and control group for the primary outcome duration of sick leave and the secondary outcome duration of sick leave starting from SSA transfer. The cases for whom the sickness benefit had not been terminated at 12 months follow-up were censored for the Cox regressions analyses. Ordinal logistic regression analysis was performed to determine the odds ratio for degree of participation between the intervention and control group. For those working during the 12-month follow-up period, the number of hours of paid employment during follow-up between the intervention and control group were compared with a linear regression model. Linear Mixed Models were used for the secondary outcomes psychological complaints and self-efficacy for RTW, with random parameters for individual baselines and fixed parameters for differential growth between the intervention and control group. Results in all analyses were adjusted for regional SSA office and type of worker (unemployed and temporary agency worker versus expired fixed-term contract worker). All analyses were performed using IBM SPSS Statistics version 25.0. In all analyses, *p*-values at or below 0.05 (two-tailed) were considered to be statistically significant.

## Results

### Recruitment of participants

**Fig. 1:**
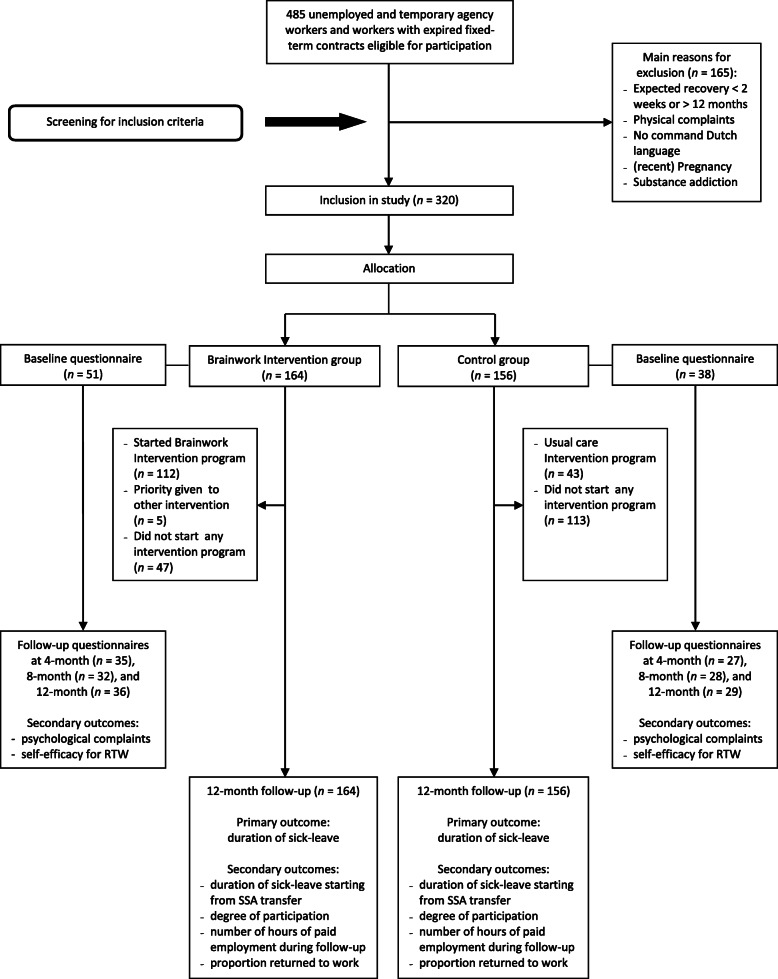
Flowchart of the study

During the recruitment period (January 2014 to September 2014), 485 potentially eligible participants were screened. Of these, 320 participants were included in the study. The Brainwork Intervention team counseled 164 participants and the usual care team 156 participants. Reasons for excluding the potentially eligible participants were: (1) expected recovery within 2 weeks of reporting sick or having contact with the vocational rehabilitation counselor of the SSA; (2) having a severe psychiatric disorder with an expected recovery later than 1 year; (3) psychological problems/complaints were not the main reason for a sickness benefit claim; (4) no adequate command of the Dutch language; (5) (recent) pregnancy; (6) substance addiction; (7) sickness claim not accepted by the SSA; and (8) not belonging to one of the three participating regional SSA offices. At baseline, 89 participants (28%) signed an informed consent to fill out the baseline questionnaire. Of these, 62 participants (19%) returned the follow-up questionnaire after 4 months, 60 participants (19%) after 8 months, and 65 participants (20%) after 12 months, for the self-reported secondary outcomes (psychological complaints and self-efficacy for RTW). Data regarding the primary outcome duration of sick leave and the secondary outcomes duration of sick leave starting from SSA transfer, proportion of workers returned to work, number of hours of paid employment during follow-up and degree of participation were available for all workers for the whole 12-month follow-up period. So all analyzes regarding the latter mentioned outcome measures involved all participants according to the intention-to-treat principle. An overview of the flowchart of the study is presented in Fig. 1.

### Baseline characteristics

Table 2 presents a summary of the baseline characteristics of the participants in the intervention and control group. At baseline there were no significant differences in characteristics of participants, and in the available characteristics of psychological complaints and self-efficacy for RTW between either group.

**Table 2:** Baseline characteristics of non-permanent workers, sick-listed due to psychological problems (*n* = 320)

	Intervention group (*n* = 164)	Control group (*n* = 156)
Age, yr (Mean ± SD)	40 ± 10.7	40 ± 10.8
Gender *n* (%) male	76 (46%)	71 (46%)
Type of worker *n* (%)		
*Unemployed/temporary agency worker*	115 (70%)	112 (72%)
*Expired fixed-term contract worker*	49 (30%)	44 (28%)
Brainwork category^a^ *n* (%)		
*Category 1*	115 (71%)	
*Category 2*	48 (29%)	
Segmentation code^b^ *n* (%)		
*Code 1*	47 (29%)	31 (20%)
*Code 2*	117 (71%)	125 (80%)
Psychological complaints (Mean ± SD)	28.4 ± 6.9	29.8 ± 6.4
*(0 to 36 score) n* = 89	*(n* = 51)	*(n* = 38)
Self-efficacy for RTW (Mean ± SD)	3.0 ± 1.03	2.6 ± 1.25
*(1 to 6 score)*) *n* = 87	*(n* = 49)	*(n* = 38)
Living area in the Netherlands *n (%)*		
East *n = 111*	58 (35%)	53 (34%)
South-west *n = 102*	52 (32%)	50 (32%)
South *n = 107*	54 (33%)	53 (34%)

^a^Brainwork category 1 = estimated recovery < 3 months

Brainwork category 2 = estimated recovery 3 to 12 months

^b^Segmentation code 1 = estimated sick leave duration < 13 weeks

Segmentation code 2 = estimated sick leave duration 13 to 52 weeks

### Adherence

In the Brainwork Intervention group, 112 out of 164 (68%) workers actually received the Brainwork Intervention program. For five workers, priority was given to another intervention. Of the 47 workers in the intervention group who did not receive any intervention, the sickness benefit of 23 workers was terminated within 2 months of the SSA receiving the sick report. In 16 workers of the intervention group (10%), at least three of the five protocol steps were followed, including timely start of the Brainwork Intervention program. In the control group, 43 out of the 156 (28%) workers received a usual care intervention. Of the 113 workers in the control group who did not receive any intervention, the sickness benefit of 16 workers terminated within 2 months of the SSA receiving the sick report.

### Primary outcome

#### Duration of sick leave

**Fig. 2:**
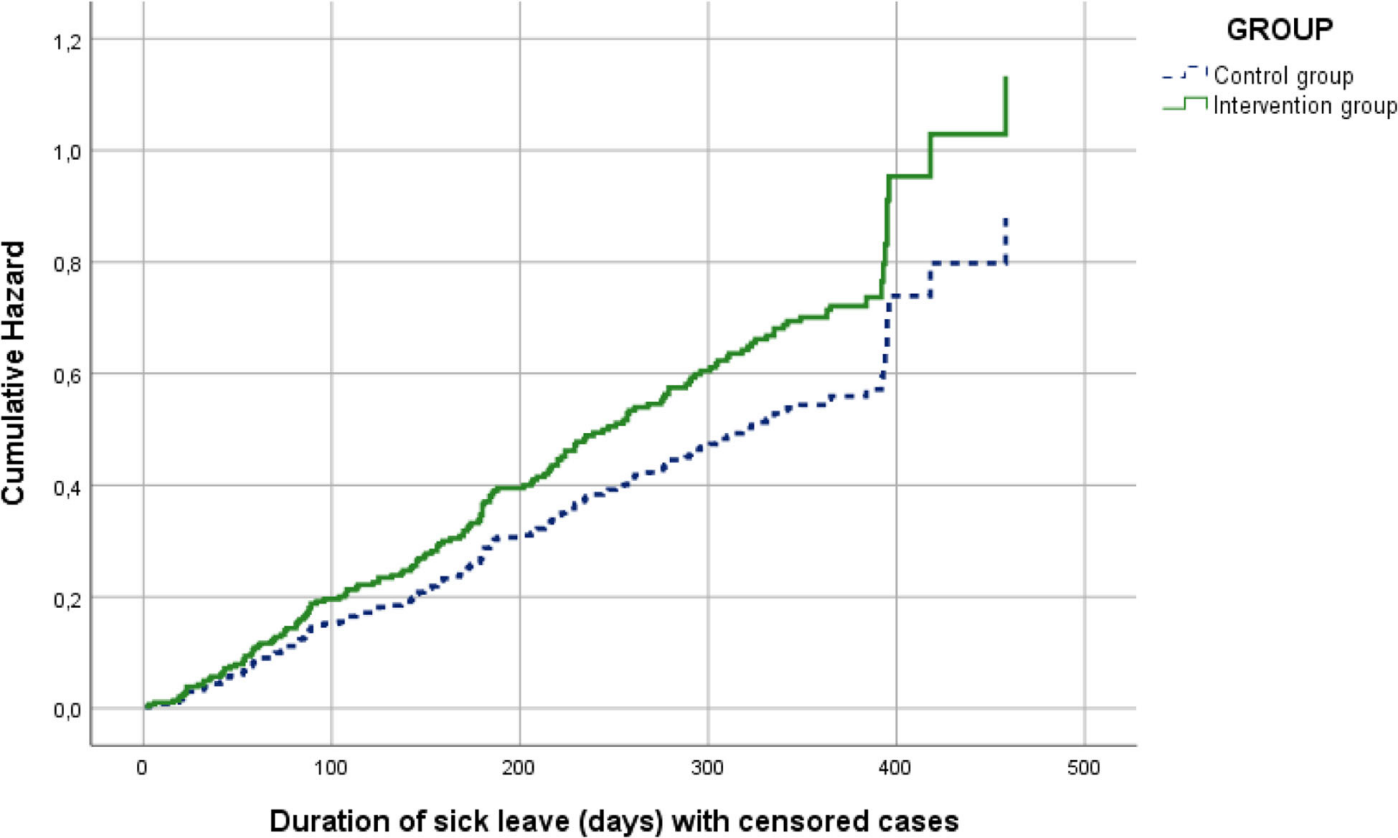
Cumulative hazard curves for the duration of sick leave in days during the 12-month follow-up for the Brainwork Intervention group and the control group adjusted for regional SSA office and type of worker

The mean duration of sick leave in the intervention group was 269 days (SD 130) versus 296 days (SD 145) in the control group, a mean difference of 27 days. The Cox regression analysis, adjusted for SSA office and type of worker, showed an HR of 1.23 (95% CI 0.94–1.76; *p* = 0.11), indicating a non-significant reduction of duration of sick leave in the intervention group compared to the control group. See Table 3 for the Cox regression results. Figure 2 shows the adjusted cumulative hazard curves for the Brainwork Intervention group and the control group. These curves show the cumulative chance for both groups that the event (termination of sick leave) occurs over time, indicating a shorter duration of sick leave in the Brainwork Intervention group**.**

**Table 3:** Cox Regression and Regression analysis results at 12-month follow-up (*n* = 320)

	Intervention group (*n* = 164)	Control group (*n* = 156)	Regression coefficient	*P*	Hazard Ratio (95% CI)
*Primary outcome*					
Duration of sick leave^a^, Mean ± SD (days)	269 ± 130	296 ± 145	0.25	0.11	1.23 (0.94–1.76)
*Secondary outcomes*					
Duration of sick leave starting from SSA transfer^a^, Mean ± SD (days)	244 ± 135	263 ± 129	0.23	0.16	1.25 (0.92–1.71)
Number of hours of paid employment during follow-up^b^, Mean ± SD (*n* = 146)	493 ± 545 (*n* = 81)	682 ± 609 (*n* = 65)	- 187.81	0.053	
Degree of participation^c^ *n* (%)					Odds Ratio^c^
*No participation*	77 (47%)	83 (53%)	- 0.27	0.24	0.77 (0.49–1.19)
*Non-paid work*	6 (4%)	8 (5%)			
*Paid work*	81 (49%)	65 (42%)			
Proportion returned to work at 12 months^d^, *n* (%) (total *n* = 161)	89 (54%)	72 (46%)			

^a^Cox regression analysis adjusted for regional SSA office and type of worker

^b^Linear Regression analysis of working participants (*n* = 146) adjusted for regional SSA office and type of worker

^c^Ordinal Regression analysis adjusted for regional SSA office and type of worker

^d^Return to work defined as end of sickness benefit

### Secondary outcomes

#### Duration of sick leave Starting from SSA transfer

**Fig. 3:**
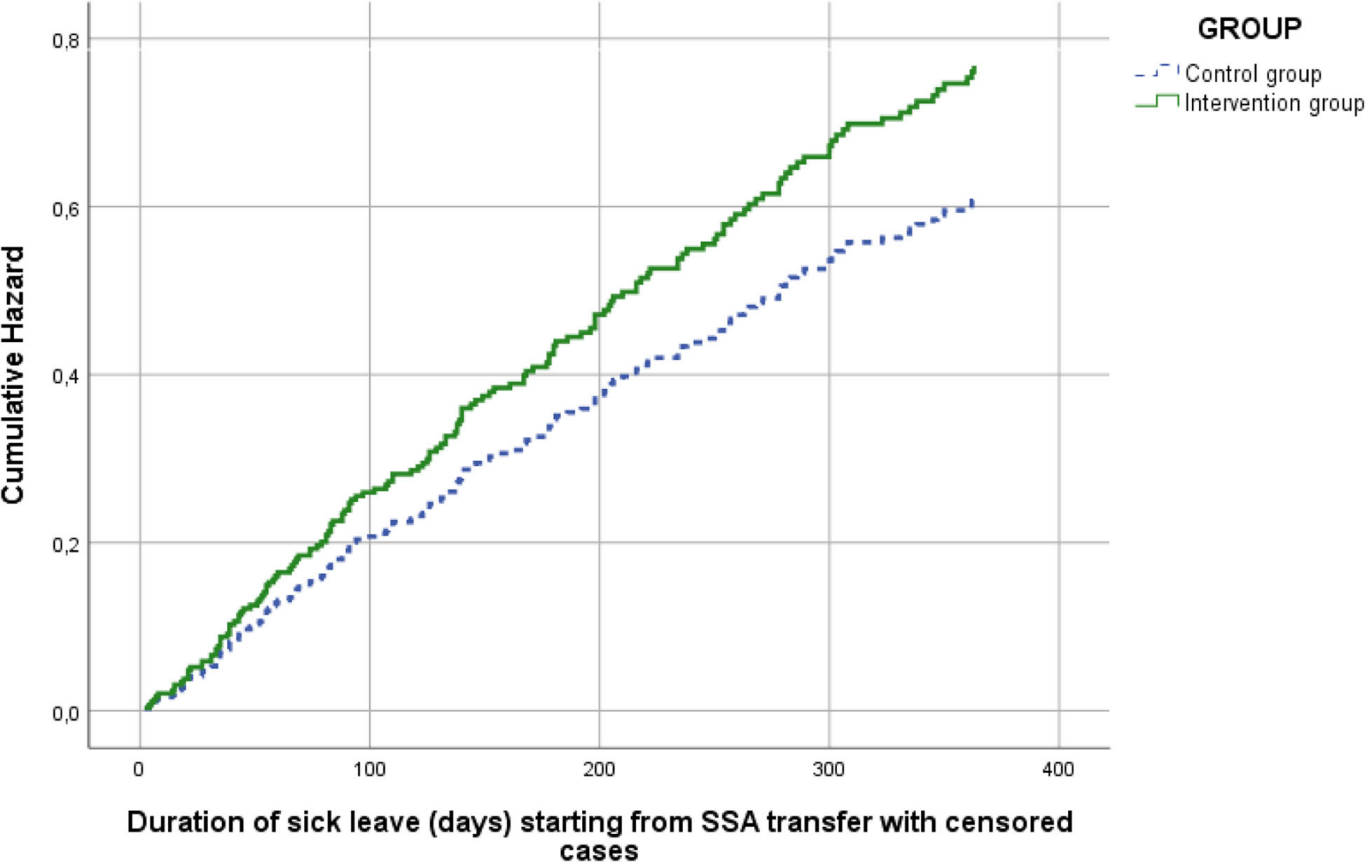
Cumulative hazard curves for the duration of sick leave in days starting from SSA transfer during the 12-month follow-up for the Brainwork Intervention group and the control group adjusted for regional SSA office and type of worker

The mean duration of sick leave starting from SSA transfer in the intervention group was 244 days (SD 135) versus 263 days (SD 129) in the control group, a mean difference of 19 days. The Cox regression analysis, adjusted for SSA office and type of worker, showed an HR of 1.25 (95% CI 0.92–1.71; *p* = 0.16), indicating a non-significant reduction of duration of sick leave starting from SSA transfer in the intervention group compared to the control group. See Table 3 for the Cox regression results. Figure 3 shows the adjusted cumulative hazard curves for the Brainwork Intervention group and control group, indicating a shorter duration of sick leave in the Brainwork Intervention group.

#### Proportion of workers returned to work

The proportion of workers returned to work (end of the sickness benefit) at 12 months follow-up after the date that the SSA received the sick report was 54% (*n* = 89) in the intervention group and 46% (*n* = 72) in de the control group. No significant differences were found between the groups.

#### Number of hours of paid employment during follow-up

During the 12-month follow-up, 81 workers in the intervention group and 65 workers in the control group had paid employment. The mean number of hours of paid employment for those working was 493 h (SD 545) in the intervention group and 682 h (SD 609) in the control group (group difference in linear regression, adjusted for SSA office and type of worker: *p* = 0.053). This indicates that for all those working, hours worked in the control group were non-significantly higher than in the intervention group. Information about contract hours was not available. See Table 3 for the results. When taking all the 320 participants into account, there were also no significant differences between the groups.

#### Degree of participation

In the intervention group 49% of the workers had paid work, compared to 42% in the control group. Ordinal regression analysis revealed an OR of 0.77 (95% CI 0.49–1.19; *p* = 0.24; control vs. intervention group), controlling for SSA office and type of worker. This indicates that participation was non-significantly lower in the control group compared to the intervention group.

#### Psychological complaints, and self-efficacy for RTW

Table 4 presents the results of the Mixed Model analyses for psychological complaints and self-efficacy for RTW, adjusted for regional SSA office and type of worker. After 12 months follow-up, although both groups showed a significant decrease in psychological complaints (adjusted mean difference − 2.85 for intervention group and − 2.61 for control group), the course of the psychological complaints between the two groups did not differ statistically (*p* = 0.76). The self-efficacy for RTW increased in both groups (adjusted mean difference 0.20 for intervention group and 0.25 for control group), but no statistical differences were found between the groups (*p* = 0.65).

**Table 4:** Results of the mixed model analyses for self-reported secondary outcomes

	Group	Baseline (T1) (*n* = 89)	4 months (T2) (*n* = 62)	8 months (T3) (*n* = 60)	12 months (T4) (*n* = 65)	*B*	Group*Time *p* value
Psychological complaints, mean (SD) (0 to 36 score)	Intervention	28.4 (6.9) (*n* = 51)	22.7 (8.6) (*n* = 35)	20.6 (8.1) (*n* = 32)	18.6 (8.8) (*n* = 36)	−2.85 (*p* < 0.01)	0.76
	Control	29.8 (6.4) (*n* = 38)	24.2 (8.5) (*n* = 27)	23.2 (8.0) (*n* = 27)	20.6 (8.5) (*n* = 29)	−2,61 (*p* < 0.01)	
Self-efficacy for RTW, mean (SD) *(1 to 6 score)*	Intervention	3.0 (1.0) (*n* = 49)	3.2 (1.28) (*n* = 35)	3.3 (1.30) (*n* = 32)	3.6 (1.38) (*n* = 36)	0.20 (*p* < 0.01)	0.65
	Control	2.6 (1.25) (*n* = 38)	2.6 (0.98) (*n* = 27)	2.9 (1.18) (*n* = 28)	3.1 (1.38) (*n* = 29)	0.25 (*p* < 0.01)	

Differences in psychological complaints and self-efficacy for RTW between the Brainwork Intervention group and the control group, adjusted for regional SSA office and type of worker

*B* = regression coefficient

## Discussion

This paper presents the effects of a newly developed Brainwork Intervention program at 12 months follow-up for non-permanent workers who were sick-listed due to psychological problems, compared to usual care. Our study indicates a non-significant reduction of the primary outcome measure duration of sick leave in the intervention group compared to care as usual. The non-significant reduction was also found for the secondary outcome measure duration of sick leave starting from SSA transfer in favor of the intervention group. Among those working during 12 months follow-up, the number of hours of paid employment during follow-up was non-significantly higher in the control group. No significant differences between the intervention and control group were found with regard to the remainder of the secondary outcomes, i.e., proportion of workers returned to work, degree of participation, level of psychological complaints and self-efficacy for RTW. Finally, the adherence to the intervention protocol was low (10%) and the tailored Brainwork Intervention was not given at all to 32% of the participants in the intervention group.

In this pragmatic controlled study, we studied the effectiveness of an expert-based intervention program developed by OH professionals of the SSA. While acknowledging the advantages of our pragmatic trial, with a good applicability of the intervention [41] and high external validity [41, 42] our design also has some disadvantages, including the low protocol adherence by professionals in the real OH setting. Furthermore, as the Brainwork Intervention is a multi-component intervention, our design does not allow us to evaluate which intervention components are responsible for failure or success. However, there are some possible explanations why the Brainwork Intervention did not show statistically significant differences between the intervention group and the control group. First, in only 10% of the participants in the intervention group were at least three of the five protocol steps followed. This means that in 90% of the participants, most of the steps of the intervention protocol, such as telephone contact with the worker, a face-to-face contact with the vocational rehabilitation counselor, or consultation of the IP, were not executed in time or were not executed at all. An explanation for the low protocol adherence on an organization level is that the work process at the participating SSA offices was not geared to such short lead times between the different steps of the intervention protocol, or due to other organizational constraints such as understaffing and a high workload. Another explanation on the behavioral level for the low protocol adherence could be that the professionals were not used to working according to a tightly prescribed protocol. Implementation research shows physicians often have problems following practice guidelines or changing their behavior to follow the guideline [43]. Although IPs mentioned that the intervention was not indicated nor necessary for some included participants, we found that in most cases (79%) there was, according to the protocol, no valid explanation or reason for not participating in the Brainwork Intervention. Furthermore we acknowledge that sick-listed non-permanent workers with psychological problems, may be a challenging group for sickness absence counseling, which may have influenced the protocol adherence. A second explanation for the non-significant results is that the intensive vocational counseling did not result in noteworthy reintegration into new primary paid work or non-paid work (placement in (temporary) workplaces), which was hypothesized as one of the essential elements of our intervention to achieve functional recovery and regain control [13, 32]. Insufficient involvement of the workplaces due to failure to find new workplaces for workers can be regarded as program failure [44]. Two other studies failed to show the effectiveness of RTW programs in sick-listed non-permanent workers and confirm that it is difficult to find workplaces (temporary or otherwise) for these workers [22, 45]. Dutch workers may also be reluctant to start a temporary new job when a sickness benefit has advantages over unemployment benefits. A recent interdisciplinary re-employment program on labor force participation among unemployed persons with common mental health problems also showed vocational counselling was not successful [23]. A third explanation for the non-significant results could be Brainwork category classification errors, which may have led to an inappropriate (i.e., lighter) Brainwork Intervention program. Brainwork category classification errors are suspected because the expected recovery time of the Brainwork category classification of the worker does not correspond to the estimated sick-leave duration of the segmentation code of the worker. Within the intervention group, the Brainwork category classification 1 (estimated recovery < 3 months) was assessed in 71% of the participants, while the segmentation code 1 (estimated sick leave < 13 weeks) was assessed in 29% of the participants (see Table 2). With an accurate assessment of the Brainwork category classification, we would expect percentages of workers with Brainwork category classification 1 to correspond more or less with the percentages of workers with segmentation code 1. Perhaps OH professionals need more training in assessing the Brainwork category classification and to achieve a better protocol adherence. The low protocol adherence and possible inappropriate assessment of the Brainwork category classification of the worker can be regarded as implementation failures. Implementation failure is a common reason for inconclusive or negative findings in intervention studies [44, 46]. A recent Dutch study by Lammerts et al. [22] evaluating a participatory supportive RTW program aimed at a comparable population, i.e., non-permanent workers with common mental disorders, also revealed implementation failure due to low protocol adherence by OH professionals of the Dutch SSA. Their explanations for this low protocol adherence were partly similar to our explanations, namely, organizational constraints (e.g., time constraints for professionals) and barriers on the behavioral level [47].

Due to the low protocol adherence (10%), relevant per-protocol analysis for the primary outcome measure duration of sick leave and secondary outcome measure duration of sick leave starting from SSA was not possible. Given the low number of 16 participants for whom the protocol was followed appropriately, the per-protocol analysis was underpowered. Further, the planned Mixed Models analyses for the outcomes regarding sick-leave duration [31] were not possible at 12 months follow-up, due to the high number of censored cases. This was because termination of sick leave had not yet occurred in more than 50% of the cases and these cases had to be censored for the analyses. We applied Cox regression analysis because this analysis technique is more appropriate for the high amounts of censored data.

The strength of this study is the complete and accurate data collection for the whole follow-up period from the SSA database for the primary outcome duration of sick leave and the secondary outcomes duration of sick leave starting from SSA transfer, proportion of workers returned to work at 12 months, number of hours of paid employment during follow-up and degree of participation. Consequently, this study has no attrition bias for these outcomes. Moreover, using registry data has the advantage of reducing the risk of detection bias in our study where sick-listed workers, occupational health professionals and intervention providers were not blinded to allocation. However, not all potential relevant baseline characteristics, e.g. educational level or marital status, were available in the registry data. A concern regarding the self-reported secondary outcomes in this study is that the response rate of the baseline questionnaire (28%) and questionnaires at 4, 8 and 12 months follow-up (19–20%) was low, resulting in the power of the study to detect changes in the self-reported secondary outcomes being low. Furthermore, a high percentage of selective non-response can bias the results if more participants with a worse mental health or longer estimated recovery period, compared to the respondents, did not return the questionnaire. However, in our study the non-response analysis with regard to the baseline characteristics and the segmentation code did not show an indication for selective non-response.

The results of our study reflect the difficulty to develop effective RTW interventions for workers with psychological complaints [48] in a SSA setting [22]. Future RTW research would benefit from a qualitative process evaluation among the involved stakeholders of the Brainwork Intervention (OH professionals, intervention partners, participants), to reveal the weakness in our research design or to explore barriers to follow the intervention protocol or to implement the intervention program activities. The information obtained from the process evaluation can provide detailed in depth sight and first hand feedback from the stakeholders, which can be used to optimize RTW interventions in the future.

Based on our study at 12 months follow-up, which had a low protocol adherence and whereby 32% of the participants in the intervention group did not receive the Brainwork Intervention at all, the use of the Brainwork Intervention program in the SSA setting cannot be recommended as a way to reduce sick leave duration. However, despite the low protocol adherence, we did find a mean difference of 27 days in favor of the intervention group. Although this difference was not statistically significant, in practice the absolute difference of 27 days is considerably and may be relevant. This difference can become probably more interesting if the adherence to the intervention protocol can be increased. IPs who intend to use interventions for non-permanent workers must realize that a range of interventions are available like Brainwork, but that little can be said about the effectiveness of these interventions. For future evaluation of RTW interventions for non-permanent workers it will be important to identify and overcome barriers for successful implementations in an early phase.

## Conclusion

We conclude that the Brainwork Intervention in the setting of the SSA and with low protocol adherence did not lead to a statistically significant reduction in duration of sick leave compared to care as usual. However, it remains unclear what the results would have been if the Brainwork Intervention had been executed according to protocol.

## Supplementary Information

**Additional file 1.** Table Brainwork category classification.

**Additional file 2.** Figure Brainwork Intervention.

## Data Availability

The datasets used and/or analyzed during the current study are available from the corresponding author upon reasonable request.

## Abbreviations

RTW: Return to work
OH: Occupational health
SSA: Dutch Social Security Agency
IP: Insurance physician
NTR: Netherlands Trial Register
GHQ-12: General Health Questionnaire-12
