# Supplementary material for: Is the “Brainwork Intervention” effective in reducing sick leave for non-permanent workers with psychological problems? Results of a controlled clinical trial
Source: BMC Public Health. 2021 Apr 9;21:698. doi: 10.1186/s12889-021-10704-0 (PMC8034165; doi:10.1186/s12889-021-10704-0)
Supplement: Supplementary file 1 — Additional file 1. Table Brainwork category classification. [file 12889_2021_10704_MOESM1_ESM.docx]

**Appendix 1:**

**Table Brainwork category classification**

| **Category 0** | **Category 1** | **Category 2** | **Category 3** |
| --- | --- | --- | --- |
| Estimated recovery  <2 weeks | Estimated recovery  <3 months | Estimated recovery  3−12 months | Estimated recovery  >12 months or unknown |
| Very mild problems | Mild psychological problems  ***OR***  Very mild problems with (severe) psychosocial problems and/or inadequate coping  ***OR***  Moderate−severe psychological problems with adequate coping | Moderate−severe  psychological problems  include somatization  ***OR***  Mild psychological problems  with (severe) psychosocial problems and/or inadequate coping style | Severe psychological problems, clinical  admission or day care treatment |
| No functional impairments | Functional impairments  (loss of control) | Severe functional impairments | Severe functional impairments to  inability for functioning |

The Brainwork Intervention is applicable for category 1 and 2
