# Supplementary material for: Is the “Brainwork Intervention” effective in reducing sick leave for non-permanent workers with psychological problems? Results of a controlled clinical trial
Source: BMC Public Health. 2021 Apr 9;21:698. doi: 10.1186/s12889-021-10704-0 (PMC8034165; doi:10.1186/s12889-021-10704-0)
Supplement: Supplementary file 2 — Additional file 2. Figure Brainwork Intervention. [file 12889_2021_10704_MOESM2_ESM.docx]

**Appendix 2:**

**Figure Brainwork Intervention**
